# Developing an emergency medicine handoff tool: an electronic Delphi approach

**DOI:** 10.1186/s12245-019-0249-4

**Published:** 2019-11-21

**Authors:** Khaled Alrajhi, Abdulmohsen Alsaawi

**Affiliations:** 1 Department of Emergency Medicine, Ministry of National Guard–Health Affairs, Riyadh, Saudi Arabia; 2 King Abdullah International Medical Research Center, King Abdulaziz Medical City, Mail Code: 1428, P.O. Box 22490, Riyadh, 11428 Kingdom of Saudi Arabia; 3 King Saud bin Abdulaziz University for Health Sciences, Riyadh, Saudi Arabia; 4 Department of Quality and Patient Safety, Ministry of National Guard–Health Affairs, Riyadh, Saudi Arabia

**Keywords:** Patient handoff, Patient safety, Emergency medicine, Delphi technique

## Abstract

**Background:**

Handoffs at the end of clinical shifts occur with high frequencies in the emergency department setting and they pose an increased risk to patients. There is a need to standardize handoff practices. This study aimed to use an electronic Delphi method to identify the core elements essential for an emergency department physician to physician handoff and propose a framework for implementation.

**Methods:**

An electronic Delphi-style study with a national panel of board-certified emergency physicians in Saudi Arabia. The panel was conducted over four rounds. The first to identify elements relevant to the end of shift handoff and categorize them into domains, while the remaining three to score and debate individual elements.

**Results:**

Twenty-five board-certified emergency physicians from various cities and practice settings were enrolled. All panelists completed the entire Delphi process. Thirty-two elements were identified and classified into 4 domains. The top five rated handoff elements were patient identification, chief complaint history, clinical stability, working diagnosis, and consulting services involved. Panel scores showed convergence as rounds progressed and the final list of elements had a high-reliability score (Cronbach’s alpha 0.93).

**Conclusions:**

This study yielded an itemized and ranked list of elements that are easy to implement and could be used to standardize patient handoffs by emergency physicians. While this study was conducted on an emergency medicine panel, the methods used may be adapted to develop standardized handoff frameworks that serve different disciplines or practice settings.

## Background

Handoffs are a daily part of the practice of emergency medicine (EM). Given the nature of shift work and the relatively high volumes of acute care visits, it is likely that emergency physicians hand off more patients than doctors in any other medical discipline [1]. In addition to handing off individual patients who are actively under the care of the outgoing physician, the emergency department (ED) end of shift handoffs often include the handoffs of some patients who are in waiting rooms, others who might be expected to arrive with ambulance services, patients who have been referred to admitting services but remain in the ED and those actively being treated in the ED. Many handover tools exist, but most are generic or have been developed for various specific disciplines or settings and therefore might not be practical or meaningful in the ED [2–5]. Techniques utilizing structured interviews and focus groups have been used to modify generic tools or create new tools for EM [6, 7]. Due to the association of handoffs with the increased risk of medical errors, both accreditation and professional agencies have called for improvement and standardization of the process [8, 9]. Nevertheless, a common standardized tool has not emerged, and handoffs remain prone to significant variations [10, 11]. The Delphi method is a well-established consensus-forming technique that has been used in various ways in the healthcare setting [12, 13]. We aimed to conduct a Delphi-style study with a panel of emergency physicians to generate and score a list of elements that may be required for the end of shift handoff.

## Methods

**Fig. 1:**
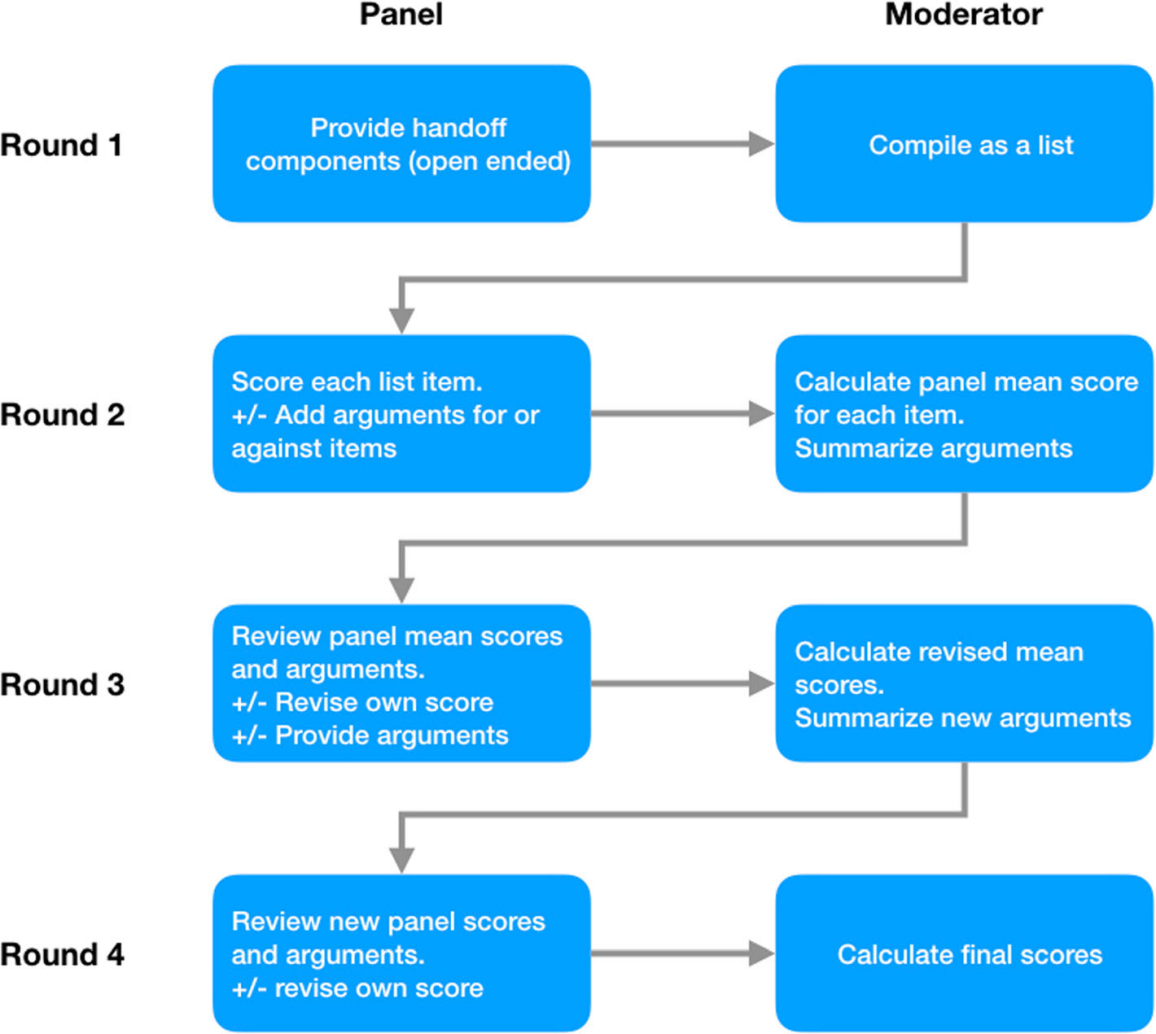
Study flow

We conducted an electronic Delphi-style study with a panel of emergency medicine consultants practicing in Saudi Arabia. At the time, this study was conducted the Saudi Commission for Health Specialties, the national licensing body, had 433 EM consultants registered. As previous literature suggests that Delphi panels with subject matter experts could be conducted with a relatively small number of experts [14, 15], we aimed for a panel of 20 to 30 consultant emergency physicians. Invitations to participate in the panel were sent to emergency physicians practicing in Saudi Arabia through the Saudi Arabian Society of Emergency Medicine. To capture providers unregistered with the Saudi Association of Emergency Medicine, open invitations were also sent to EM attending physicians through group emails and social media sites. All invitations contained a link to a web-based form to register as a panelist. To mitigate the loss of panelists during the study period, the form’s introduction detailed the expected panel process, including what would be expected of panelists and the amount of time that might be required. Panelists were required to be board-certificate in EM with no fewer than 3 years in practice as an EM attending physician. The Delphi process had 4 rounds (Fig. 1). In the first round, panelists were asked to provide itemized lists of all elements that they considered relevant for an end of shift handoff. The moderator compiled the lists, removed duplicates and organized the elements into general domains. In round 2, the compiled list of elements was sent to panelists in an electronic form for every panelist to score each element. As most elements provided by the panelists could be very important in any given specific situation, panel members were instructed to not score items on importance alone but rather on how frequently each element was required during handoffs, from 1 “rarely required” to 10 “always required.” Panelists were also able to add comments to support their positions. In round 3, each panelist received a spreadsheet showing the group’s average scores compared to the panelist’s own score for each element, along with the compiled comments or arguments shared by other panelists. Each panelist would then have a chance to change their score and/or comment on the group’s score to influence other panelists. In round 4, the average scores were recalculated based on the previous round’s results, and the comments were compiled and sent to each panelist in a spreadsheet similar to the one used in round 3; the panelists were given a final chance to revise their scores. Panel members were anonymous and unaware of each other’s identities throughout the study. The moderator, an emergency physician, did not contribute input to the panel. Emails and phone calls were used to communicate with panelists for follow up on missing input or clarifications on comments. Google forms, Google sheets, and Microsoft Excel spreadsheets were used to collect scores and comments in rounds 2 to 4.

The mean score for each element and pooled standard deviations for all elements were used as measures of central tendency and spread. We calculated the absolute difference in each element’s mean score between rounds and summed the differences to assess the amount of change in panel scores between rounds as a measure of the panel’s stability. The intraclass correlation coefficient (ICC) using a two-way random-effects model for consistency was also used to assess the panelist agreement in every round [16, 17]. Cronbach’s alpha was used to assess the reliability of the itemized list of handoff elements.

Statistical Package for Social Sciences (SPSS for Mac, Version 21; SPSS, Inc., Chicago, IL) was used for the analysis.

## Results

Registration was open for 6 weeks in early 2018. Twenty-nine EM physicians completed the registration form, and 4 were excluded because they had fewer than 3 years of experience as an EM attending physician. The 25 included panelists practiced in 16 different hospitals in 6 cities in Saudi Arabia, with an average of 7.9 years of post-board certification experience. All four rounds of the study were completed by the panel concluded over a period of 6 weeks. All panelists completed all four rounds of the study, and there were no dropouts.

Thirty-two specific handoff elements were identified and rated according to the described Delphi methods. Those were placed into the following four domains: nonclinical patient information, clinical patient information, patient course in the ED, and general ED status. The general ED status domain includes elements that are either general to the department or not specific to an individual patient being handed off. At the conclusion of the panel, the individual element mean scores ranged from 4.7 to 9.8 out of a maximum of 10 (Table 1). The top five rated elements were the chief complaint history, patient identification, clinical stability, working diagnosis, and consulting services involved.

**Table 1:** Final panel scores

Domain	Element	Round 2		Round 3		Round 4	
		Mean	SD	Mean	SD	Mean	SD
Nonclinical patient information	Patient identification (combination of name, age, and medical record number)	9.12	1.39	9.24	1.27	9.4	0.87
	Location	8.72	2.11	8.52	1.58	8.4	1.58
	Eligibility for treatment or insurance status	5.72	3.09	4.92	2.63	4.7	2.56
Clinical patient information	Ambulatory or chair/bed bound	6.20	2.75	5.60	2.10	5.3	1.82
	Code status	8.40	2.45	7.56	2.45	7.5	2.33
	Chief complaint history	9.68	0.75	9.80	0.50	9.8	0.50
	Relevant past history	8.92	1.75	8.60	1.19	8.6	1.23
	Home medications	6.64	2.66	6.00	2.42	5.8	2.30
	Allergies	8.60	2.45	7.80	2.87	7.8	2.46
	Vital signs	8.80	1.87	8.20	1.78	8.0	1.88
	Physical exam findings	8.76	1.56	8.24	1.42	8.2	1.33
	Working diagnosis	9.56	0.96	9.24	1.20	9.2	1.20
	Clinical stability (e.g., stable, borderline, or unstable)	9.64	0.76	9.40	0.96	9.2	0.97
Emergency department course	Summary of the results of investigations	9.28	1.90	8.52	2.18	8.4	2.16
	Investigations ordered but pending	8.72	2.28	8.00	2.24	8.0	2.09
	Treatments given	9.40	1.15	9.04	1.40	9.0	1.29
	Changes in condition in the ED	9.36	1.52	8.72	1.81	8.4	2.06
	Consulting services involved	9.44	0.92	9.48	0.77	9.4	0.77
	Likely disposition plan (home, admission or consultation)	8.76	1.67	8.44	1.64	8.5	1.48
	Alternate plan (when changes in condition, the results or discussion with consultants are likely to alter the original plan)	7.44	2.35	6.80	2.14	6.8	2.06
	Identify parts of the plan that need to be completed by the incoming team	8.60	1.94	7.80	1.89	8.0	1.58
	What has been discussed with the patient or their family?	7.56	2.75	7.12	2.09	7.0	1.93
	Identify patients who is being handed over but need to be seen as a new patient (e.g., incomplete assessment or challenging presentation)	9.44	1.08	9.24	1.30	9.2	1.35
Emergency department status	Identify high-risk patients who may have been referred to other services or are still waiting to be seen	9.08	1.78	8.64	1.93	8.8	1.61
	Identify patients who require isolation	8.88	1.99	8.52	2.14	8.5	2.14
	Conflicts with patients or families	7.64	2.53	6.68	2.32	6.6	2.24
	Conflicts or delays with consulting services	8.52	2.08	7.88	2.05	7.8	1.55
	Waiting status (number of patients, waiting time and acuity)	6.84	2.79	6.68	2.29	7.0	1.99
	Beds status (e.g., boarding, isolation, and critical care beds)	7.68	2.51	7.44	2.18	7.4	1.80
	Patients expected to arrive via ambulances or referred from clinics or other facilities	7.88	2.33	7.00	2.10	7.0	1.86
	Shortages in medications, equipment, or supplies	7.44	3.25	6.80	3.07	6.7	2.88
	Shortages in staffing	7.84	2.87	7.56	2.52	7.4	2.48

*SD* standard deviation

The sum of absolute differences of elements means between rounds 2 and 3 was 15.8. Between rounds 3 and 4, the sum of absolute differences was only 3.48. The pooled standard deviations for all element scores in rounds 2, 3, and 4 were 2.1, 1.9, and 1.8, respectively. The panelists ICC for rounds 2, 3, and 4 were 0.23, 0.30, and 0.36, respectively. Reliability testing for the elements returned a Cronbach’s alpha of 0.93, 0.89, and 0.93 for rounds 2, 3, and 4 respectively.

## Discussion

This study demonstrates the use of the Delphi method in developing a discipline-specific set of handoff elements that could be utilized in the development of a handoff tool. Handoff tools have been commonly developed using focus groups or unstructured interviews (e.g., I-PASS [6, 18] and ABC [7]), but formal consensus development methods appear to have rarely been used for this purpose. The Delphi process is a formal consensus forming method that offers some key advantages for approaching handoffs in a specialized field [9]. Focus groups and brainstorming sessions are ideal for idea generation, but specialty experts may not require exploratory or idea-generating techniques as much as they require the prioritization of ideas that are typically shared and well known within the discipline. While group interviews and focus groups allow for better articulation of opinions, they may be dominated by stronger individuals or coalitions and the open format may inhibit some members from speaking freely [9]. They are also difficult to organize with larger groups or with members in different geographical areas. In contrast, the electronic Delphi is most practical for a relatively large group of subject matter experts who may not be able to meet in a specific time or location and the anonymity of Delphi panelists helps limit undue influence exerted by members who may be more senior in rank or more outspoken. In both I-PASS and ABC studies, the outcomes of staff interviews were thematic and non-discrete and needed to be further modified or refined by the facilitators into practical tools ready for implementation. In comparison, the Delphi method we describe yields a discrete list of items by design and the results could be directly used in a handoff tool when needed.

One of the limitations of the electronic Delphi method is the lack of face-to-face interactions that allow for groups to clarify ideas and shared understandings. This was noticed when some panelists on occasions gave an element a lower score only because they felt it was redundant and should be considered part of another element, while others considered the element at face value and scored it independently. Our design did not allow the group to revise the summarized list of elements produced after round 1. It is possible that an additional round of voting to select elements that needed to be broken down into several individual elements or individual elements that could have been combined might have added some value.

This study did not use a uni-dimensional scoring scale but rather a scale that combines the elements importance and frequency of use. Instead of asking panelists “how important is this element?” and “how frequently is this element used?”, we asked panelists about how frequently would a given element be on the important end of the scale “How frequently is this element required?”. While it could be argued that each of the two factors should have its own scale, we believe that the result of such an exercise would likely include elements with low importance and high frequency and others that are rare but important. Such a list is not readily usable and is impractical to implement as it would require further input from yet another subject matter expert to decide the weight and ranking of each factor. We opted to offload this cognitive exercise onto the panel of experts who, as a group, were most likely to be capable of balancing all factors involved in scoring elements in their own domain of expertise. This was supported by the high-reliability coefficient of the survey. However, much of the panel’s discussion revolved around how relevant a given element is in a handoff. Some elements may be critical in a given scenario but less important in another (e.g., “vital signs” in a critically ill patient vs. a young patient with a twisted ankle). A scoring scale that revolves around the element’s relevance (i.e., from “required only when directly relevant” to “required even when remotely relevant”) might offer a solution and may be explored in future studies.

Furthermore, with regard to the scoring structure, the frequency an element is required in a handoff is heavily influenced by the setting. It is possible that this might have skewed the overall scores towards the higher end of the scale because most of the panelists practiced in tertiary care teaching hospitals where more patients with more complex conditions require the provision of more details during handoffs (e.g., code status).

The panel’s ICCs were low, but this was expected given the relatively large number of panelists (25) and elements (32), leading to 800 assessments for each round. The intent was to examine the trend of the ICC measurements as rounds move forward. As rounds progressed, the panel’s ICC continued to increase showing increasing agreement on elements. The absolute sum of differences of the means and the pooled standard deviations both show clear convergence in round 4, also showing improved consensus.

Handoff tools serve as checklists to help reduce human errors. Although many handoff tools have been studied in healthcare settings [5, 6], most are generic and lacking in specificity and may not always be meaningful for providers working in a setting with specific expectations in a patient handoff. This study design addresses this issue by yielding a set of items specific to the discipline of the panelists. It also has practical appeal as the outcome’s format is easy to implement in checklists or forms on paper or electronic systems. It is also easy to replicate with different disciplines and on various scales from a single department within an institution to international professional groups and scientific societies.

**Fig. 2:**
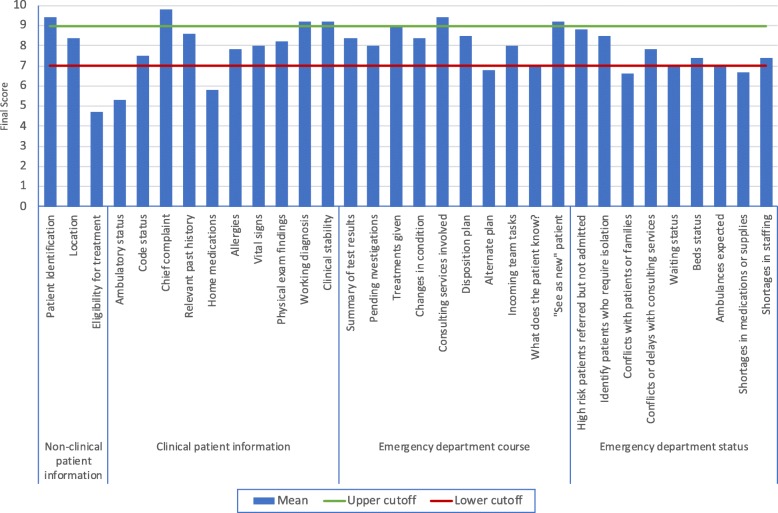
A proposed final outcome of the handoff of an individual ED patient with a proposed lower cutoff line for inclusion of elements in a handoff tool and an upper cutoff line for mandatory elements

We propose that the outcomes of a study conducted with the methods presented be used as a framework for developing standardized handoff tools. Professional groups may choose arbitrary cutoff values to design a handoff tool based on the results of such studies. These cutoff values could help decide which elements to include or which elements may be mandatory in a proposed handoff tool. Cutoff values may also be chosen on the basis of acuity for the area or department the handoff tool is intended for so that providers would have different handoff tools for different settings. For the purposes of illustration, we proposed upper and lower cutoff values for the elements in our study to facilitate a potential implementation in an electronic health record. In this example, elements above the upper cutoff value would be mandatory fields for all handoffs, those between the two cutoff values would be optional based on the context, and elements below the lower cutoff value would be excluded from the form (Fig. 2).

## Conclusions

This study demonstrates how the electronic Delphi method was used in the development of an emergency medicine end-of-shift handoff tool using a panel of subject matter experts located in different locations and time zones. While the specific elements in this study may or may not be suitable for general use, the process yields an itemized and ranked list of elements that is easy to adopt on paper or electronic forms should it be implemented.

## Data Availability

The datasets used and/or analyzed during the current study are available from the corresponding author on reasonable request.
